# Individual and Environmental Correlates to Quality of Life in Park Users in Colombia

**DOI:** 10.3390/ijerph14101250

**Published:** 2017-10-19

**Authors:** Diana Marina Camargo, Paula Camila Ramírez, Rogério César Fermino

**Affiliations:** 1 Research Group in Harmony, Movement and Life, Faculty of Physical Therapy, Universidad Industrial de Santander, Bucaramanga 680002, Colombia; pcramire@uis.edu.co; 2 Research Group in Human Being, Culture and Movement, Universidad Santo Tomás, Bucaramanga 680001, Colombia; 3 Research Group in Environment, Physical Activity and Health (GPAAFS), Postgraduate Program in Physical Education, Federal University of Technology-Parana, Curitiba-PR 81310-900, Brazil; rogeriofermino@utfpr.edu.br; 4 Research Group in Physical Activity and Quality of Life (GPAQ), Pontiff Catholic University of Parana, Curitiba-PR 80215-901, Brazil

**Keywords:** quality of life, built environment, parks, recreational, health status

## Abstract

*Purpose:* To explore individual and environmental correlates to quality of life (QoL) in park users in Colombia. *Methods:* A cross-sectional study with face-to-face interviews was conducted with 1392 park users from ten parks in Colombia. The survey included sociodemographic questions, health condition assessed with EuroQuol-5-Dimensions-5-Levels; in addition, questions about accessibility to the parks and perceptions about quality of infrastructure and green areas were asked. The Spanish version of the questionnaire EUROHIS-QOL-8 items was applied to assess QoL. Log-binomial regression models were applied for analyses. *Results:* Years of schooling, visits to the park with a companion, active use of the park, a maximum score for quality of trees and walking paths, and the perception of safety on the way to the park were positively associated with a better QoL (*p* < 0.05). Health conditions related to problems in the ability to perform activities of daily living and anxiety/depression showed negative associations. *Conclusions:* The present study contributes to the Latin American studies by providing information on how parks in an intermediate city may contribute to increased QoL of park users through safety in neighborhoods, social support, active use, and aesthetics, cleanliness, and care of green areas.

## 1. Introduction

In 1994, the World Health Organization (WHO) defined quality of life (QoL) as “individuals’ perceptions of their position in life, in the context of the culture and value systems in which they live and in relation to their goals, expectations and concerns.” [1]. This definition emphasizes a subjective and multi-dimensional concept because it includes several aspects such as health, psychological status, degree of independence, relationships with other people and with the environment, and spiritual or religious beliefs [1]. 

The growing level of urbanization worldwide makes the quality of cities and the built environment relevant in defining quality of life of their citizens [2]. In the environmental context, parks are identified as green spaces for collective use that act as regulators of environmental balance and represent an element of the natural heritage. They are designed for recreation, contemplation, and leisure of the citizens [3,4]. Parks also contribute to the conservation of natural resources and the ecosystem, reduce pollution, generate equality in inhabitants’ development, contribute to the preservation of historical memory and create a sense of identity in the communities [3]. Besides, parks enhance the population’s active lifestyles, mental health, and social cohesion while decreasing exposure to toxic conditions [3,5]. The availability of open spaces to people (such as appealing and safe parks) contributes to more active behavior in the population, social responsibility, and care of the public resources. It also improves coexistence, tolerance, health, and QoL of inhabitants [3,6].

The literature on associations between the use of parks and quality of life is scarce. From an ecological level, Larson [7] established a positive yet not significant relationship between park quantity (measured as the percentage of city area covered by public parks), park quality (measured as per capita spending on parks), and accessibility (measured as the overall percentage of a city’s population within ½ mile of parks). The study suggests that increasing these public spaces contribute to health and well-being in cities and positively impact urban quality of life.

Recently, Koramaz [8] proposed a conceptual model to understand the relationships between urban parks and health, physical activity, social relations, satisfaction with the neighborhood and their contributions to improve quality of the residential environment. The purpose of this model is to create a healthy society, especially in densely developed residential areas, giving clues into policies for increasing QoL through planning and design interventions for residential environments and urban parks [8].

Efforts to preserve open spaces and to create green infrastructure on a larger scale could be useful in making a positive impact on public health and the population’s QoL [5,9]. For this reason, accessibility to these spaces in terms of proximity, walkability, travel time, safety, shared activities, and available leisure time among other factors should be a priority when deciding to invest in land conservation and parks [10,11]. Some studies assess the relationship between perceptions of natural environment infrastructure and one or more aspects of QoL. Positive associations were found for mental health in university students [12] and older adults [13]. Furthermore, a walk of 150 min or more during the previous week and the safety of the public space were positively associated with perceived health among senior citizens in North America [14]. 

In Colombia, two previous studies found the relevance of the built environment and parks for human well-being and quality of life. However, both highlight the need to expand the evidence in this regard, considering the role of some culturally distinctive features of people, the level of mass urbanization, and differences in the levels of connectivity, density, and land-use mix [15,16]. The aim of this study was to explore potential associations between individual and environmental correlates of QoL in Colombian park users.

## 2. Materials and Methods 

### 2.1. Location Of the Study and Ethical Aspects

Bucaramanga is an intermediate city located on a plateau at 959 masl in the Cordillera Oriental (Easter Mountain Range) of the Colombian Andes. It has a municipal area of 165 km^2^ (approximately 64 square miles), a mean temperature of 23 °C (73.4 °F), and 529,785 inhabitants. With a population density of 3130 hab/km^2^ and public space of 4.53 m^2^/hab, 52.4% of the homes in Bucaramanga are houses, 40.4% apartments, and the remaining 7.2% corresponds to other buildings. Socioeconomic status (SES) is determined by using the classification from the city Planning Department, which has six categories from 1 to 6 (poorest to richest), distributed as: 1–2 (29%), 3–4 (60.1%), 5–6 (10.9%) [17].

Traditionally, Bucaramanga has been recognized as “The city of parks”. From a total of 140 parks, there are 10 metropolitan parks (≥25 Ha), 10 zonal parks (1.5–25 Ha), 83 local parks (0.1–1.5 Ha), 37 pocket parks (<0.1 Ha) and an index of green space of 2.51 m^2^/hab [18]. 

Like many Latin American cities, Bucaramanga’s road and urban infrastructures have been growing, but with disorderly planning which makes the movement of vehicles and pedestrians difficult. In addition, the small area of the city on the plateau has caused priority to be given to the building of large-scale works for housing and mobilization, causing stress and discomfort for people. 

The Ethics Committee of the Universidad Industrial de Santander approved this project in the Act number 12 of 23 May 2014. All participants signed an informed consent form before being enrolled in the study.

### 2.2. Design

This is a cross-sectional study conducted in two zonal and eight local parks of the city. The selection criteria were: access (*the community has free access to the park because of a non-existent or partial enclosure*); operation and function (*the park accomplishes the role described by the Land Use Plan (Plan de Ordenamiento Territorial)* [17] *for local parks (Free sports, games for children and adults, active and passive recreation with equipment, areas for free sports)* and safety conditions during the inspection visit. 

### 2.3. Data Collection

Parks were visited once in each selected day of the week (Tuesday, Wednesday, Thursday, Saturday, and Sunday) and users were surveyed face-to-face. Users were defined as people of both sexes, older than 12 years, who stayed in the park for at least 15 min they were selected by non-probabilistic sampling. Members of the fieldwork team interviewed them and each interview took between 12 and 15 min. Data collection was conducted between September and December 2015. Two observation periods were conducted from 6:00 am to noon and from 3:00 pm to 8:00 pm. The period between noon to 3:00 pm was not observed, based on cultural custom, when people go home for lunch and rest. Also, people usually take a nap before going back to work at two o´clock in the afternoon, reducing the number of park users; additionally, it is the time of the day with the highest temperatures. 

#### 2.3.1. Explanatory Variables 

The following parameters were evaluated by a questionnaire designed for this study with 24 items: Sociodemographic variables (sex, age, length of residency in the neighborhood, city of residency, marital status, years of schooling, and number of children); Accessibility (visits to parks in the last month and year, visits to parks by proximity to home, frequency of visits, time in minutes from home to park, walk to get to the park, company for park visit, and active versus passive use). Active use was defined as walking, working out, or engaging in any kind of physical activity that involves movement, such as playing with a ball, jogging, walking a pet, walking with children/grandchildren, other, while passive use was sitting on a bench, just leaving the house, reading, chatting with friends, going for a drink, eating, other [19].

Park visitors were also asked nine items about their perception of some safety features and infrastructure of the park. This includes the condition of green areas, trees, and paths for walking and jogging on a scale from 1 (very low) to 5 (excellent), with the absence of the feature scored as zero.

Self-perception of health status was measured using the Spanish version of the EuroQol-5 Dimensions-5 Levels (EQ-5D-5L) questionnaire [20], a standardized measure of health status developed by the EuroQol Group in order to provide a simple, generic measure of health for clinical and economic appraisal. It includes five dimensions: mobility, self-care, usual activities, pain/discomfort, and anxiety/depression. Each attribute was scored on a 5-point ordinal scale, ranging from zero (0): “I have no problems…, I have no pain…, I am not anxious…”, to four (4): “I am unable to…, I have extreme pain…., I am extremely anxious…”. A visual numeric scale (0–100 mm) with endpoints labeled with descriptive sentences, from “the worst health you can imagine” to “the best health you can imagine,” is also included.

#### 2.3.2. Outcome Variable

The QoL was measured using EUROHIS-QOL 8 questionnaire [21]. It is composed of eight items (overall QoL, general health, energy, daily life activities, self-esteem, relationships, finances, and household). The overall QoL score is formed by a simple summation of scores on the eight items, with higher scores indicating better QoL. All answer scales have a 5-point response format on a Likert scale, ranging from ‘not at all’ to ‘completely’. The use of this questionnaire was approved by the World Health Organization.

A pilot test was done with 30 park users to standardize the procedures and determine the clarity of the questionnaire. We evaluated the internal consistency of the EUROHIS-QOL-8 questionnaire (Cronbach’s α coefficient = 0.83) and the reliability for each item (Kappa 0.33–0.64), also the total score showed an Intraclass Correlation Coefficient of 0.69 (95% CI, 0.44–0.84). 

Based on responses from the 1392 park users, we applied a Rasch analysis to the questionnaire, which showed a good fit after recoding to the original scores from 12345 to 00012. The reliability results were for items (1.0) and persons (0.77); separation index for items (15.5) and persons (1.85). The INFIT and OUTFIT ranges varied between 0.74–1.30 and 0.73–1.25, for items and persons respectively. The explained variance was 46.3% and the unexplained variance in the first contrast was 11.2%. A differential item functioning was not detected for either sex, age, schooling or active use of the park. Finally, the output variable was generated from the measurement obtained by the Rasch Model, that did not show a normal distribution, so a dichotomous variable was created from the median value as the cut-off point for the multivariate analysis. 

### 2.4. Data Analysis

The associations were assessed by applying simple and multiple log-binomial regression models, to estimate the prevalence ratios (PR) and their 95% confidence intervals. The modeling process followed Greenland’s recommendations [22]. Considering the cluster sampling of the park users in the 10 parks studied, simple and multiple log-binomial regression analyses were adjusted by sampling method. The analyses were done in STATA 14.1 (Stata Press, 4905 Lakeway Drive, College Station, TX, USA) at a 5% significance level.

## 3. Results

A total of 1392 users participated in the study (response rate of 95%), median age 42 years (range: 12–86), 813 (58.4%) women, 510 (36.6%) married (Table 1).

Ninety four percent of people visited the park during the previous year and 58.8% with a companion; 59% declared an active use of the park (Table 2). 

In perception of safety, 28.5% considered dangerous going to the park, 69.5% always feel safe on the way to the park, and 71.5% feel safe within the park.

The scores of the global condition for park infrastructure features are shown in Table 3. The higher scores by park users were registered for trees and walking paths with 43.1% and 35.6%, respectively.

Health status items (Table 4), reflect a good overall assessment of self-care and usual activities. However, the dimensions of pain, anxiety/depression, and mobility showed the lower prevalence in this category (75%). Additionally, the visual numeric scale showed a median value of 90 (range 0–100).

The scores for EUROHIS-QOL 8-items showed a high level of satisfaction (4 and 5) for all dimensions, except for having enough money for daily needs (Table 5); the overall QoL score registered a median value of 33 (range 13–40).

Years of schooling, visiting the park with a companion, active use of the park, a maximum score (5) for the condition of trees and walking paths, and the perception of safety on the way from home to the park were positively associated with a better QoL (Table 6). Only health conditions related to problems in the ability to perform daily living activities and the expression of anxiety/depression showed negative associations. Sex, age, length of residency in the neighborhood, and frequency of park visits did not show significant associations in the simple regression analysis (*p* > 0.25) and were not included in the final model. 

## 4. Discussion

This is the first study in Colombia and Latin America that explores some individual and environmental correlates to perceived QoL in park users. Findings are supported in the theoretical model proposed by Koramaz [8] in which the interaction between subjective and objective personal and environmental characteristics perceived by persons related with urban parks, social relationships, health, and physical activity contribute to their QoL. From this point of view, it is a useful resource to guiding concerted interventions by authorities, planners, and users to improve their quality and services.

User characteristics found in this study are similar to those found in Brazil [23], where 63% of users were female; in the United States of America (USA) [24] and Canada [25] there were more men, 62% and 64%, respectively. These differences could be attributed to the number of facilities and size of parks available for the community in North American countries. Furthermore, the weather conditions in cities like Bucaramanga and Recife, near to the equator, may also make a difference in the preferences of their inhabitants for park visits.

This study showed a prevalence higher than 90% for visits to parks during the past year and month, which may be explained by closeness to home (72.6%), time from home to park (median 10 min) and walking as transport to the park (74%), and frequency of visit of more than once a week (74.1%). Fifty-nine percent of people visited the park with a companion and the same prevalence was registered for active use of parks. Also, the positive perception of safety was reported by 70% of the users. These results could be explained by the theoretical model proposed by Bedimo-Rung et al. [3], Koramaz et al. [8], and Wang et al. [11] in which personal characteristics of the users, social support by friends and family and health condition, aggregated to physical characteristics of the environment like safety and aesthetics, could contribute to differential uses of parks and QoL of users.

The availability of local and pocket parks in Bucaramanga offer safe and nearby spaces to the residents of the neighborhood that, despite having a smaller area compared to those in the USA and Canada [24,25], has been identified as one of the main factors associated with their use. In Curitiba, 61% of people visited a park [26] and walking was the favorite activity of the users [27]. Also, each additional use per week of the natural environment reduced the risk of poor mental health by 6%, underlining the importance of the natural environment, in which forests and parks seem to have a more beneficial effect [28].

There were no studies associating years of schooling and QoL in park users. However, it is possible to explain that education affects the quality of life of individuals in many ways. People with low schooling have fewer opportunities of high-paying jobs. Moreover, low-income neighborhoods are evident in its characteristics of safety, cleanliness, public services, and public spaces. These environmental characteristics could correlate in a negative way with their well-being and QoL [29].

Among the perceptions of environmental factors associated to better QoL, safety on the way to the park (PR = 1.17), visiting the park with a companion (PR = 1.17), and highest scores for trees and walking paths (PR = 1.25 and 1.18, respectively) were significant. The effects of physical built environments like parks on some dimensions of QoL have been reported in several studies, at any age group. 

Canadian adolescents reported increased emotional well-being due to the perception of neighborhood safety and decreased due to the perception of trash and abandoned houses [30]. For university students, the interference of the natural environment on their mental health is explained by their preferences for known environments, and they point to the benefit of mature trees and water sources which provide an opportunity for relaxing and reflecting off-campus [12]. For older adults, green and blue spaces are an important component of maintaining habits and QoL. Without a work routine, older adults need to devote time to activities that provide a daily routine and give them reasons to leave the house reducing boredom, isolation, and loneliness and giving their lives physical, spiritual, and social purposes, important dimensions of QoL [14]. 

The positive role of size, aesthetics, and quality of public and green spaces on QoL have been demonstrated in several studies. Van Den Berg [31] concluded that contact with nature in parks has a positive association with overall health, particularly for low-strata groups and the immigrant population, contributing to feelings of well-being and relaxation; Song [32] demonstrated by experimental design, that walking in parks compared to streets generates a better mood as well as less anxiety and fewer negative emotions; Grinde [33] established that aesthetics and nature are very important in generating a positive effect on the well-being and health of people, giving pleasure to the mind, stress relief, mental restoration and mood improvement in conscious or unconscious ways. 

Therefore, environmental aspects are central to urban planning in developing countries, especially given the limited public and green space available, as well as the high level of urbanization in the coming years, which could reflect in detriment of air quality, physical activity, and health of citizens [34]. A few limitations of the study must be pointed out. First, this was a cross-sectional study, so no causal associations can be made; second, we assessed all research items through a face-to-face survey and only park users were surveyed, which could represent potential reporting and selection biases; third, this is an exploratory study that included only some variables potentially associated to QoL from the individual and environmental point of view of park users.

However De Jong et al. [35] showed correlations between 0.32 and 0.51 when comparing subjective versus objective measurements for assessing the green space of neighborhoods, which could support a potential control of a classification bias in the measurement performed via questionnaire versus objective measurements. In addition, conceptual models and observational studies support the associations found in this study.

## 5. Conclusions

The present study contributes to the Latin American studies by providing information on how the parks in an intermediate city may contribute to increased QoL of park users through safety in neighborhoods, social support, active use, aesthetics, cleanliness, and care of green areas. These findings must engage urban planners and decision makers to increase the quantity and quality of public space, planning programs, and policies to protect, create, and promote the natural environment as parks for citizens. 

## Figures and Tables

**Table 1:** Socio-demographic characteristics of park users (N = 1392).

Variable	Male N (%) 579 (41.6)	Female N (%) 813 (58.4)	Total N (%) 1392 (100)
**Age (years) *Median (IQR)***	46 (29–58)	41 (27–54)	42 (28–55)
**Age group *n (%)***			
12–20	35 (6.6)	50 (6.8)	85 (6.7)
21–59	368 (70.0)	572 (77.9)	940 (74.6)
≥60	123 (23.4)	112 (15.3)	235 (18.6)
**Marital status *n (%)***			
Married	227 (39.2)	283 (34.8)	510 (36.6)
Single	190 (32.8)	246 (30.3)	436 (31.3)
Cohabitant	113 (19.5)	158 (19.4)	271 (19.5)
Other	49 (8.5)	126 (15.5)	175 (12.6)
**City of residence *n (%)***			
Bucaramanga	528(91.2)	751 (92.4)	1279 (91.9)
Floridablanca	28(4.8)	32 (3.9)	60 (4.3)
Girón/Piedecuesta/Other	23 (4.0)	30 (3.7)	53 (3.8)
Length of residency (years) in the neighborhood *Median (IQR)*	10 (3–23)	7 (2–20)	8 (2–20)
**Years of Schooling *n (%)***			
1–6	98 (17.4)	152 (19.1)	250 (18.4)
7–9	51 (9.0)	75 (9.4)	126 (9.3)
10–12	189 (33.4)	281 (35.3)	470 (34.6)
13–14	92 (16.3)	129 (16.2)	221 (16.2)
≥15	135 (23.9)	158 (19.9)	293 (21.5)
**Children *n (%)***			
Yes	418 (72.1)	652 (80.2)	1070 (76.9)
**Number of children *n (%)***			
1	91 (21.7)	196 (30.1)	287 (26.8)
2	142 (34.0)	224 (34.4)	366 (34.2)
≥3	185 (44.3)	232 (35.6)	417 (39.0)

**Table 2:** Characteristics of the visit to the park by the park users (N = 1392).

Variable	Men N (%) N: 579	Women N (%) N: 813	Total N (%) N: 1392
Visited parks within the past month (YES)	525 (90.7)	728 (89.5)	1253 (90.0)
Visited parks within the past year (YES)	554 (95.7)	755 (92.9)	1309 (94.0)
Visited parks because it is close to home	414 (71.5)	596 (73.3)	1010 (72.6)
Visited the park two or more times during a regular week	442 (76.3)	590 (72.6)	1032 (74.1)
Time (minutes) from home to park *Median (IQR)*	7 (5–15)	10 (5–15)	10 (5–15)
Walking as transport to park *n (%)*	412 (71.0)	619 (76.0)	1031 (74.1)
Usually visits the park with a companion	268 (46.3)	551 (67.8)	819 (58.8)
Park use			
Active	378 (65.3)	444 (54.6)	822 (59.0)
Passive	201 (34.7)	369 (43.4)	570 (41.0)

**Table 3:** Perceptions related with global condition of park infrastructure features (N = 1392).

Variable	Score ^a^					
	0 N (%)	1 N (%)	2 N (%)	3 N (%)	4 N (%)	5 N (%)
Overall cleanliness and hygiene	1 (0.07)	74 (5.3)	138 (9.9)	367 (26.4)	467 (33.6)	344 (24.7)
Meadows and green areas	27 (1.9)	236 (17.0)	268 (19.3)	350 (25.2)	299 (21.5)	211 (15.2)
Trees	1 (0.07)	34 (2.4)	87 (6.3)	222 (16.0)	447 (32.1)	600 (43.1)
Walking paths	4 (0.3)	26 (1.9)	61 (4.4)	233 (16.8)	572 (41.1)	495 (35.6)
Jogging trails	207 (14.9)	24 (1.7)	61 (4.4)	226 (16.3)	495 (35.6)	378 (27.2)
Bicycle routes	1102 (79.2)	21 (1.5)	34 (2.4)	73 (5.3)	92 (6.6)	69 (5.0)

^a^ Score: 0: Absent/1: Very low/2: Low/3: Moderate/4: Good/5: Excellent.

**Table 4:** Health status of the park users evaluated through the EQ5D-5L questionnaire (N = 1392).

Dimension	Score N (%)				
	0 N (%)	1 N (%)	2 N (%)	3 N (%)	4 N (%)
Mobility ^a^	1233 (88.9)	81 (5.8)	52 (3.7)	20 (1.4)	3 (0.2)
Self-care ^a^	1342 (96.6)	30 (2.2)	11 (0.8)	6 (0.4)	0 (0)
Usual activities ^a^	1322 (95.2)	1 (3.0)	19 (1.4)	6 (0.4)	0 (0)
Pain/discomfort ^b^	1041 (75.0)	167 (12.0)	125 (9.0)	52 (3.7)	4 (0.3)
Anxiety/depression ^c^	1215 (87.5)	121 (8.7)	39 (2.8)	12 (0.9)	2 (0.1)

^a^ Score: 0: I have no problems/4: I am unable. ^b^ Score: 0: I have no pain or discomfort/4: I have extreme pain or discomfort. ^c^ Score: 0: I am not anxious or depressed/4: I am extremely anxious or depressed.

**Table 5:** Quality of life of the park users evaluated through EUROHIS-QOL 8-items.

Dimension	Score				
	1 N (%)	2 N (%)	3 N (%)	4 N (%)	5 N (%)
How would you rate your quality of life ^a^	2 (0.1)	33 (2.4)	184 (13.3)	822 (59.2)	347 (25.0)
How satisfied are you with your health? ^b^	2 (0.1)	41 (3.0)	130 (9.3)	774 (55.8)	441 (31.8)
Do you have enough energy for everyday life? ^c^	1 (0.3)	35 (2.5)	175 (12.6)	599 (43.2)	535 (38.5)
How satisfied are you with your ability to perform your daily living activities? ^b^	2 (0.1)	25 (1.8)	107 (7.7)	719 (51.8)	441 (31.8)
How satisfied are you with yourself? ^b^	0 (0.0)	23 (1.7)	81 (5.8)	605 (43.6)	679 (48.9)
How satisfied are you with your personal relationships? ^b^	6 (0.4)	29 (2.1)	138 (9.9)	659 (47.5)	556 (40.1)
Do you have enough money to meet your needs? ^c^	18 (1.3)	112 (8.1)	591 (42.6)	447 (32.2)	220 (15.8)
How satisfied are you with the conditions of your living place? ^b^	9 (0.6)	77 (5.6)	144 (10.3)	581 (41.9)	577 (41.6)

^a^ Score: 1: Very bad/5: Very good. ^b^ Score: 1: Very unsatisfied/5: Very satisfied. ^c^ Score: 1: Not at all/5: A lot.

**Table 6:** Associated factors to quality of life in park users (N = 1357, Link test *p*: 0.18).

Variable	Simple Regresion PR ^a^	95% CI	Multiple Regresion PR ^a^	95% CI
	Not Adjusted		Adjusted	
Years of schooling				
1–6	1.0		1.0	
7–9	1.31	0.96–1.78	1.25	0.96–1.62
10–12	1.48	1.15–1.90	1.37	1.09–1.71
13–14	1.49	1.05–2.12	1.36	0.96–1.92
≥15	1.58	1.18–2.11	1.51	1.16–1.97
Visit to the park with a companion	1.16	1.00–1.34	1.12	1.01–1.25
Active use of the park	1.13	0.96–1.33	1.14	1.00–1.30
Tree conditions status (score = 5)	1.22	1.05–1.42	1.20	1.07–1.34
Walking paths conditions (score = 5)	1.33	1.10–1.61	1.21	1.03–1.41
Perception of safety on the way from home to the park (always)	1.26	1.10–1.44	1.22	1.04–1.44
Health status (EQ5D)				
Slight problems in walking to unable to walk (1–4)	0.19	0.07–0.50	0.39	0.17–0.90
Slight to extreme Anxiety/Depression (1–4)	0.61	0.48–0.76	0.69	0.54–0.86

^a^ PR: Prevalence Ratio.
